# Comparison of Endostar continuous versus intermittent intravenous infusion in combination with first‐line chemotherapy in patients with advanced non‐small cell lung cancer

**DOI:** 10.1111/1759-7714.13106

**Published:** 2019-06-03

**Authors:** Yuan Cheng, Ligong Nie, Ying Liu, Zhe Jin, Xi Wang, Zhanwei Hu

**Affiliations:** ^1^ Department of Respiratory and Critical Care Medicine Peking University First Hospital Beijing China

**Keywords:** Continuous intravenous infusion, Endostar, NSCLC

## Abstract

**Background:**

Intravenous infusion of Endostar for three to four hours per day for 14 days reduces patient compliance and affects quality of life. Continuous intravenous infusion (CI) represents a novel method of administration; however, it is unclear whether it is effective and safe when compared to the traditional method.

**Methods:**

We retrospectively reviewed patients with advanced non‐small cell lung cancer (NSCLC) administered CI (20 patients) or intermittent intravenous infusion (II, 49 patients) of Endostar combined with first‐line chemotherapy. Three patients in the II group discontinued therapy because of adverse effects.

**Results:**

Median progression‐free survival was 6.0 months in the CI group and 3.8 months in the II group, with no significant difference (*P* = 0.1). The objective response and disease control rates were also similar in the CI and II groups (40.0 vs. 32.6%, *P* = 0.562; 65 vs. 69.6%, *P* = 0.714, respectively).

**Conclusion:**

CI of Endostar combined with first‐line chemotherapy for advanced NSCLC had similar progression‐free survival, objective response, and overall response rates as II, with tolerable adverse effects.

## Introduction

Folkman first proposed the vascular dependence of tumor growth in 1971.^1^ Angiogenesis is the result of a dynamic imbalance between proangiogenic and antiangiogenic factors. Vascular endothelial growth factor (VEGF) and its receptors are important factors in tumor angiogenesis. Antiangiogenic drugs have a synergistic effect when combined with chemotherapy. Endostatin is a carboxyl terminal proteolytic fragment of collagen XVIII, which acts as an endogenous inhibitor of angiogenesis. Endostar is a novel modified recombinant human endostatin, and was approved by the Food and Drug Administration of China for the treatment of non‐small cell lung cancer (NSCLC) in September 2005. Endostar can normalize tumor vasculature, inhibits tumor angiogenesis, endothelial cell proliferation and migration via downregulating a number of angiogenic factors including VEGF.^2^, ^3^, ^4^ In clinical trials of Endostar in combination with traditional chemotherapy for advanced NSCLC, Endostar significantly improved the outcome in patients, demonstrating an antitumor effect.^5^

Traditional Endostar administration is intermittent intravenous infusion for three four hours per day for 14 days; however, long‐term treatment reduces patient compliance and affects quality of life. In this study, two administration strategies were compared in terms of efficacy and safety without adjusting the total dosage in order to provide more clinical data for the treatment of patients with advanced NSCLC.

## Methods

We retrospectively reviewed the records of patients with advanced NSCLC who received at least two cycles of Endostar in combination with first‐line chemotherapy at Peking University First Hospital from April 2010 to October 2018. The inclusion criteria were: (i) histological diagnosis of NSCLC; (ii) unresectable stage IIIA, IIIB or IV (as defined by the American Joint Committee on Cancer Tumor Node Metastasis staging system version 7.0); (iii) an Eastern Cooperative Oncology Group (ECOG) performance status (PS) score of 0–1; and (iv) at least one measurable lesion according to Response Evaluation Criteria in Solid Tumors (RECIST), version 1.1. The ethics committee of Peking University First Hospital approved this study, and all patients signed informed consent.

### Treatment

The patients were administered Endostar in combination with first‐line chemotherapy. The patients were divided into intermittent intravenous infusion (II) and continuous intravenous infusion (CI) groups according to the different methods of administration. In the II group, Endostar was administered daily at a dose of 7.5 mg/m^2^/day in 500 mL of saline for four hours from days 1 to 14. In the CI group, Endostar was continuously pumped at a rate of 10 mL/hour (105 mg/m^2^ in 1000 mL of saline) via a mini‐osmotic pump from days 1 to 5.

### Evaluation of efficacy and safety

Efficacy was evaluated by computed tomography (CT) scan after every two cycles according to RECIST version 1.1, including complete response (CR), partial response (PR), stable disease (SD), and progressive disease (PD). The disease control rate (DCR) was defined as the percentage of patients with CR, PR, and SD. The overall response rate (ORR) was defined as the percentage of patients with CR and PR. PFS was considered as the time from diagnosis to tumor progression or death from any cause. Adverse events were classified using National Cancer Institute Common Terminology Criteria for Adverse Events, version 4.0.

### Statistical analysis

Statistical analysis was performed using SPSS version 23.0. All of the categorical variables, ORRs, DCRs, and incidences of adverse effects were analyzed and compared between the groups using the *X*^2^test. PFS curves were drawn using the Kaplan–Meier method and GraphPad prism version 7.0. *P* < 0.05 was considered statistically significant.

## Results

### Patient characteristics

Sixty‐nine patients met the inclusion criteria and were included in the analysis. A total of 49 patients received Endostar II and 20 received CI. Three patients in the II group discontinued therapy because of adverse effects. The baseline characteristics and demographics were similar between the groups (Table 1). The median ages were 58.5 and 62 years in the II and CI groups, respectively. All patients received platinum‐based chemotherapy.

**Table 1:** Patient characteristics

Characteristics	II group	CI group	Total	*P*
Age, years (median)		33–78 (58.5)	39–77(62)	0.383
Gender				0.375
Male	37	13	50	
Female	12	7	19	
ECOG PS				0.851
0	33	13	46	
1	16	7	23	
Histological type				0.16
Squamous carcinoma	12	9	21	
Adenocarcinoma	29	7	36	
Other	8	4	12	
Stage				0.223
III	3	4	7	
IV	40	14	54	
Postoperative relapse	6	2	8	
Pleural effusion				0.707
No	17	6	23	
Yes	32	14	46	
Chemotherapy				0.547
Gemcitabine plus platinum	34	13	47	
Pemetrexed plus platinum	13	7	20	
Paclitaxel plus platinum	2	0	2	
Heart disease history				0.486
No	44	19	63	
Yes	5	1	6	
Hypertension history				0.675
No	39	15	54	
Yes	10	5	15	
Smoking history				0.333
No	16	9	25	
Yes	33	11	44	

CI, continuous intravenous infusion; ECOG PS, Eastern Cooperative Oncology Group performance status; II, intermittent intravenous infusion.

**Table 2:** Response to treatment in II and CI groups

Response	II Group (*n* = 46)	CI Group (*n* = 20)	*P*
PFS (months)	3.8	5.95	0.1
PR	15	8	
SD	17	5	
PD	14	7	
ORR%	32.6%	40.0%	0.562
DCR%	69.6%	65.0%	0.714

CI, continuous intravenous infusion; DCR, disease control rate; II, intermittent intravenous infusion; ORR, overall response rate; PD, progressive disease; PFS, progression‐free survival; PR, partial response; SD, stable disease.

### Efficacy analysis

**Figure 1:**
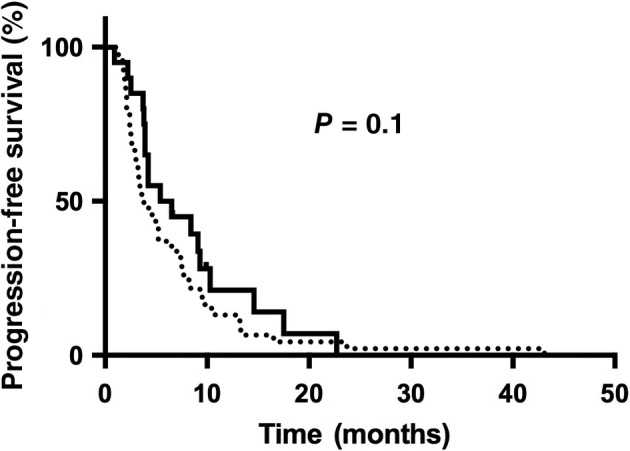
Kaplan–Meier estimates of progression‐free survival. CI, continuous intravenous infusion; II, intermittent intravenous infusion.

Three patients were excluded from efficacy analysis because they discontinued Endostar after the first cycle of treatment. None of these patients achieved CR. Fifteen and 8 patients achieved a PR in the II and CI groups, respectively (Table 2). Seventeen cases of SD were observed in the II group and five in the CI group. Figure 1 shows the Kaplan–Meier curve for overall PFS. The median PFS was 6.0 months in the CI group and 3.8 months in the II group, with no significant difference (*P* = 0.1). The ORRs between the CI and II groups were not significantly different (40.0 vs. 32.6%, respectively; *P* = 0.562). The DCR in the CI group was also similar to that in the II group (65 vs. 69.6%, respectively; *P* = 0.714).

### Safety

Three patients in the II group discontinued therapy as a result of adverse effects: deep vein thrombosis (1 patient), skin rash (1 patient), and atrial fibrillation (1 patient). The incidence rates of all drug‐related adverse events were 70% in the CI and 81.6% in the II group, with no significant difference (*P* = 0.288). The incidence rates of drug‐related grade 3 or 4 adverse events were 50% in the CI group and 36.7% in the II group. There were no significant differences between the groups (*P* = 0.309). The common adverse events observed in the groups are summarized in Table 3. The incidence rates of myocardial ischemia were 10 and 0% in the CI and II groups, respectively, with a significant difference between the groups (*P* = 0.025). Two patients with myocardial ischemia presented with mild myocardial enzyme elevation without chest pain or other related symptoms. No change was observed on electrocardiogram.

**Table 3:** Treatment‐related adverse events

	All adverse events (%)			Grade 3 or 4 (%)		
Adverse event	II group (*n* = 49)	CI group (*n* = 20)	*P*	II group (*n* = 49)	CI group (*n* = 20)	*P*
Any	40 (81.6)	14 (70)	0.288	18 (36.7)	10 (50)	0.309
Hematological toxicity						
Granulocytopenia	24 (49)	8 (40)	0.497	12 (24.5)	5 (25)	0.964
Anemia	8 (16.3)	2 (10)	0.498	3 (6.1)	1 (5)	0.856
Thrombocytopenia	9 (18.4)	2 (10)	0.389	6 (12.2)	2 (10)	0.792
Non‐hematological toxicity						
Arrhythmia	5 (10.2)	0	0.138	1 (2.0)	0	0.52
Myocardial ischemia	0	2 (10)	0.025	0	0	
Nervous system disorder	2 (4.1)	0	0.359	0	0	
Rash	3 (6.1)	1 (5)	0.856	0	0	
Transaminase elevation	0	2 (10)	0.025	0	0	
Vomiting	5 (10.2)	1 (5)	0.486	0	0	
Infection	1 (2.0)	1 (5)	0.506	0	1 (5)	0.115
Nausea	6 (12.2)	1 (5)	0.366	0	0	
DVT	2 (4.1)	2 (10)	0.34	1 (2.0)	1 (5)	0.506
Hemorrhage	0	1 (5)	0.115	0	1 (5)	0.115

CI, continuous intravenous infusion; DVT, deep vein thrombosis; II, intermittent intravenous infusion.

## Discussion

In animal models, the antitumor activity of Endostar is more preponderant than bevacizumab, while they show comparative antiangiogenic effects.^6^ The traditional method of Endostar administration reduces patient compliance and affects quality of life. CI is a novel method of administration performed 24 hours a day through a small intravenous infusion without changing the total dose. The half‐life of Endostar is 10 hours, and studies have shown that CI delivery reduces the toxicity of the drug, prolongs the retention time of the drug in the blood, increases the active ingredient in the target tissue, and induces tumors apoptosis by stabilizing the blood concentration of the drug.^7^ Hansma *et al*. studied the safety of CI at different doses, and indicated that this route of delivery is safe.^7^ Chen *et al*. showed that there was a linear correlation between the exposure of the body to Endostar and the administered dose between 7.5 and 30 mg/m^2^/day.^8^ The HELPER study showed that CI of Endostar combined with concurrent chemotherapy and radiotherapy had similar PFS, prolonged OS, and tolerable toxicities compared to other studies.^9^

The median PFS rates of the CI and II groups in this study were 6.0 and 3.8 months, without significant difference. The ORR and DCR rates were also similar, indicating that the efficacy of CI was similar to that of II. We also examined whether toxicity is altered when the total drug administration time is significantly shortened. There were no significant differences in any grade or grade 3–4 side effects or hematological toxicity between the groups. Meta‐analysis showed that the use of angiogenesis inhibitors is associated with higher rates of hypertension, thromboembolism, and cardiac ischemia.^10^ Based on the phase III trial, cardiovascular related events are the most notable toxic reaction, with an incidence rate of approximately 4.8% in myocadial ischemia and 8.1% in arrhythmia.^5^ In our study, the incidence rates of arrhythmia and myocardial ischemia were 0 versus 10.2% (*P* = 0.138) and 10 versus 0% (*P* = 0.025) in the CI and II groups, respectively. One of the three patients had a history of hypertension, but none had a history of coronary heart disease. The incidence rate of myocardial ischemia is statistically significant, suggesting that CI administration may cause minimal myocardial damage, but it seems to be unrelated to previous cardiovascular disease.

The mechanism of myocardial damage from antiangiogenic treatment has not been extensively investigated, although hypotheses as to an underlying off‐target pathophysiologic mechanism of cardiotoxicity have been proposed.^11^ The most important consideration in regard to interaction with other chemotherapeutics is the very likely additive adverse action on endothelial cells. While VEGF is expressed in the normal myocardium, the consequences are most likely revealed when its expression is upregulated as part of a healing or compensation response, and it is under such circumstances that most cases of cardiotoxicity occur.^12^ Therefore, it is necessary to closely observe and monitor cardiac toxicity during Endostar administration. The cardiotoxicity of Endostar is reported to be slight and reversible;^13^ however, close observation of the heart rate, electrocardiogram, myocardial enzymology markers, and cardiac ultrasound of patients during such therapy is recommended.

There are some limitations to this study. First, as a retrospective rather than a prospective study, there are certain limitations. Second, a small number of cases, particularly the CI sample, were enrolled, which can lead to bias and affect various factors and the statistical results. As few studies of the cardiotoxicity of the continuous intravenous infusion method of administration have been conducted, further research is needed.

In conclusion, CI of Endostar combined with first‐line chemotherapy therapy for advanced NSCLC yielded similar PFS, ORR, and DCR to II, with tolerable adverse effects. Prospective randomized studies are warranted to further evaluate treatment response.

## Disclosure

No authors report any conflict of interest.
